# Preparation and Characterization of a Biodegradable Film Using Irradiated Chitosan Incorporated with Lysozyme and Carrageenan and Its Application in Crayfish Preservation

**DOI:** 10.3390/foods12142642

**Published:** 2023-07-08

**Authors:** Liang Qiu, Qinghua Luo, Chan Bai, Guangquan Xiong, Shiwei Jin, Hailan Li, Tao Liao

**Affiliations:** 1 Hubei Engineering Research Center for Agricultural Products Irradiation, Institute of Agro-Products Processing and Nuclear Agricultural Technology, Hubei Academy of Agricultural Sciences, 5th Nanhu Avenue, Wuhan 430064, China; ql9202@hbaac.com (L.Q.); chanbai@haac.com (C.B.); guanquanx69@haac.com (G.X.); haill85@haac.com (H.L.); 2 Key Laboratory of Cold Chain Logistics Technology for Agro-Product, Ministry of Agriculture and Rural Affairs, Wuhan 430064, China; 3 Key Laboratory of Catalysis and Energy Materials Chemistry of Education, Hubei Key Laboratory of Catalysis and Materials Science, South-Central University for Nationalities, Wuhan 430074, China; ql38438@hotamail.com (Q.L.); shiweijin@stun.edu.com (S.J.)

**Keywords:** chitosan, irradiation modification, biodegradable film, crayfish preservation

## Abstract

In this study, a composite film was prepared using irradiated chitosan, lysozyme, and carrageenan for crayfish preservation. First, the chitosan was degraded by gamma rays, with the best antimicrobial properties being found at 100 KGy. By using the response surface method, the components of the composite film were irradiated chitosan (CS) at 0.016 g/mL, lysozyme (LM) at 0.0015 g/mL, and carrageenan (CA) at 0.002 g/mL. When compared to the natural chitosan film, the Fourier-transform infrared spectroscopy (FTIR) and X-ray diffraction (XRD) results demonstrated that the chemical properties of the composite film did not change with the addition of LM and CA, while the physical and antibacterial properties increased, including tensile strength (16.87 → 20.28 N), hydrophobicity (67.9 → 86.3°), and oxygen permeability (31.66 → 24.31 m^3^·um/m^2^·day·kPa). Moreover, the antibacterial activity of the films increased with the addition of LM and CA, especially for *Shewanella putrefaciens*: the zone of inhibition (mm) of CS, CS/LM, and CS/LM/CA was 9.97 ± 0.29, 14.32 ± 0.31, and 14.78 ± 0.21, respectively. Finally, the CS/LM/CA film could preserve crayfish for 10 days at 4 °C, whereas the polyethylene (PE) film could only preserve them for 6 days. Moreover, the composite film was excellent at inhibiting oxidative deterioration (TBARS value: 2.12 mg/kg, day10) and keeping the texture of crayfish muscle. Overall, our results suggested that the CS/LM/CA composite film produced can be applied as a biodegradable film in aquatic product packaging.

## 1. Introduction

Aquaculture, especially for crustaceans such as crayfish (*Procambarus clarkii*), is an essential economic pillar industry and an increasingly important component of the global food supply [1,2,3]. However, aquatic products containing rich nutrition and high water content can be easily spoiled due to temperature fluctuations and environmental effects during transportation and storage [4,5]. Thus, it is important and urgent to develop efficient preservation methods that can maintain aquatic product quality and food freshness [6].

Packaging materials play a very important role in the preservation process. At present, the commonly used packaging materials are polyethylene (PE), polydichloroethylene (PVDC), and polyvinyl chloride (PVC) due to their low costs and good characteristics, such as tensile strength and heat sealability [7]. However, these plastic materials are difficult to degrade, which can cause serious ecological pollution and increase the environmental burden over the long term. Biodegradable packaging materials, such as those based on polysaccharides and proteins, can avoid this issue because of their edibility and reproducibility, among other properties. Therefore, how to produce biodegradable packaging materials, such as films and wrappers, has attracted increasing attention [8,9,10].

Chitosan (CS) is a natural carbohydrate with the advantages of biodegradability, nontoxicity, and biocompatibility, which has been widely used in agriculture, materials science, and environmental protection [11]. However, the low antioxidant and poor physical properties of natural chitosan, such as solubility and film forming, limit its application in packaging materials [12]. The antioxidant activity of chitosan increases with decreasing molecular weight since the ammonium (NH^3+^) ion at the C_2_ position stabilizes free radicals by providing H^+^ ions [13]. At present, the degradation methods of chitosan include chemical breakdown [14], enzyme catalysis, the use of ultrasonic waves [15], and other physical methods [16]. Among these methods, chemical and enzymatic methods produce many byproducts, and their processes are difficult to control [17]. Irradiation modification has become a successful method in materials science, with the advantages of being a simple process and having a low cost, along with high purity and no secondary pollution [18]. For example, Rahman et al. reported that irradiated chitosan could increase the growth rate of Malabar spinach and significantly inhibit fungal growth [19]. Thus, using irradiation-modified chitosan as a basis to produce packaging materials has good application prospects [20,21].

CS-based packaging materials that are composited with natural macromolecules have improved mechanical and film-forming properties, such as improved phenolic and polyphenolic contents [22]. κ-carrageenan can improve the physico-mechanical properties of chitosan films due to the opposite ionic charges between the chitosan layers, resulting in decreased water vapor permeability when compared to single-layer chitosan films [23]. Sangbin Kimet et al. reported that κ-carrageenan/chitosan nanolayered coatings exhibited lower oxygen permeability when compared to chitosan or ι-carrageenan film [24]. Lysozyme, also known as N-acetyl cytoplasmic glycan hydrolase or cytoplasmic enzyme, is a glycoside hydrolase that specifically acts on the cell wall of micro-organisms [25]. Researchers have used lysozyme in combination with chitosan for its antibacterial characteristics [26]. Chong et al. used different concentrations of lysozyme, chitosan, and calcium chloride to produce a fresh-keeping solution for strawberry preservation. The composite film extended the shelf life of strawberries for up to 14 days [27].

Therefore, the objective of this study was to elaborate a new food packaging material by incorporating two fillers from different sources into irradiated chitosan film (CS). We selected γ-rays to modify chitosan to keep the process as green as possible. Besides, κ-carrageenan (CA) and lysozyme (LM) were chosen because of their natural ingredients while having the ability to improve the physical and antibacterial activity properties of films. These ingredients were added using different concentrations into a chitosan film via a casting method, and their effect on the physicochemical, barrier, and functional properties of the polymer film was evaluated. In addition, since the formulation contains only biocompatible and environmentally friendly ingredients, a food preservation test was developed for crayfish to evaluate the possibility of using this material for food packaging.

## 2. Materials and Methods

### 2.1. Materials

Live crayfish with an average weight of 30 ± 2.4 g and an average length of 20 ± 1.8 cm were purchased from Qianjiang Changgui Aquatic Food Co., Ltd. (Nantong, China). Four strains of bacteria, including *Escherichia coli* (AB93154), *Shewanella putrefaciens* (ATCCBAA-1097), *Staphylococcus aureus* (ATCC43979), and *Salmonella typhimurium* (ATBA45479), were purchased from the Shanghai Preservation Biotechnology Centre, (Shanghai, China). All other reagents (chitosan, lysozyme, carrageenan, MgO, KBr, trichloroacetic acid, etc.) used in this study were analytically pure and were purchased from McLean Biochemical Technology Co. Ltd. (Shanghai, China).

### 2.2. Irradiation Degradation of Chitosan

Chitosan was sent to the Hubei Irradiation Experimental Center for ^60^Co γ-ray irradiation. The dosage rate was 6.25 Gy/min, and the irradiation doses were set at 20, 40, 60, 80, and 100 KGy [28]. Then, we used a Euclidean viscometer to determine the relative viscosity of the chitosan and calculate the molecular weight. Meanwhile, bacteriostatic experiments using *Escherichia coli*, *Shewanella putrefaciens*, *Staphylococcus aureus*, and *Salmonella typhimurium* were carried out with irradiated chitosan at different doses, and the growth curve was determined based on absorbance using an ultraviolet spectrophotometer (UV-3600i Plus, Shimadzu, Tokyo, Japan).

### 2.3. Preparation of Films

First, pure acetic acid was added to deionized water to create a 1% acetic acid solution, and then irradiated chitosan was added. To fabricate the CS/LM and CS/LM/CA films, lysozyme and κ-carrageenan were separately added to the CS solution. All the film-forming solutions were sonicated for 120 min at room temperature (40 KHZ, J100S, Sonic, Shenzheng, China) and then stirred at 25 °C for 30 min using a magnetic stirrer (60 rpm, C-MAGHS 4, IKA, Baden, Germany). All the films were prepared using the casting method: 15 mL of the solution was cast into a Petri dish (plastic plate, 90 mm) and then put into the oven at 45 °C, with the baking time set for 6–8 h. Afterward, the Petri dish was taken out and cooled to room temperature, and the film was peeled off and placed at 25 °C with 50% relative humidity for storage.

### 2.4. Experiment Design

For our single-factor experiments, the concentration range of the compounds used is listed in Table 1. A response surface experiment was designed, and the best compound ratio of CS, LM, and CA was determined.

### 2.5. Thickness and Mechanical Properties of the Films

The thickness of the films was measured using a digital display vernier caliper (NISCO Company, Tokyo, Japan); six random points were obtained each time, and the average was taken. The mechanical properties were determined based on the ASTMD882 standard [29] with some modifications. The films were cut into 5 cm × 1.5 cm long strips before testing, and the tensile test program was selected using the TMS-Pro texture analyzer (Stable Micro System Ltd., Godalming, UK) for measurement. The set parameters were as follows: the initial nip distance was 20 mm, and the tensile rate was 60 mm/min. Six strips were measured in each group of samples, and the average of these results was taken. The tensile strength (*T_S_*) and elongation break (*EB*) were calculated according to the following formulas:

(1) $$T_{S}=F/A$$

(2) $$EB=\frac{L_{1}-L_{0}}{L_{0}}\times 100\%$$

where *F* is the maximum tension at fracture in N; *A* is the cross-sectional area in mm^2^; *L*_0_ is the original length in mm; *L*_1_ is the length at fracture in mm; *T_s_* is the tensile strength in MPa, and *EB* is the elongation at break (%).

### 2.6. Morphology and Color of the Films

After the films had dried, a colorimeter (Konica Minolta Sensing Inc., Osaka, Japan) was used to determine the color of the films according to a previously reported method [30]. The colorimeter adopted an 8° directional illumination diffusion-receiving method with a measurement aperture of 8 mm. All measurements were conducted under a standard D65 light source and 10° observation conditions. Five measurements were made at random locations on each film, and the L* (brightness), a* (red-green), and b* (yellow-blue) values were recorded. The whiteness (W) value was calculated as follows:

(3) $$W=100-\sqrt{{\left(100-L*\right)}^{2}+{a*}^{2}+{b*}^{2}}$$

### 2.7. Water Vapor Transmission Rate (WVTR) and Oxygen Permeability

Based on the previously reported methods with minor modifications [31], each composite film was cut into a circle with a diameter of 100 mm and sealed in a test cup with CaCl_2_, and then the cup was placed in a dryer (25 °C and 50% relative humidity). The cup was weighed every 12 h for 10 days until reaching a constant weight, and each sample was triple replicated. The water vapor transmittance of the film was calculated, as shown in Formula (4):

(4) $$WVTR=\frac{\Delta m\cdot d}{A\cdot t\cdot \Delta p}$$

where Δ*m* is the weight change of the cup (g); *d* is the mean film thickness (mm); *A* is the area of exposed film (m^2^); *t* is the time interval (24 h), and Δ*p* is the water vapor pressure difference (kPa) on both sides of the film.

The methods for measuring the oxygen permeability of the films were based on previous studies [32]. A pressure-difference gas permeation instrument was used to detect the oxygen permeability of the CS, CS/LM, and CS/LM/SA films. The diameter of the circular sample used for testing was 80 mm, the pressure difference was 10^5^ Pa, and the test temperature was 23 °C.

### 2.8. Water Contact Angle

The contact angle of the films was measured using a contact angle analyzer (Krüss, Hamburg, Germany) and the sessile drop method [33]; continuous shooting using dual focus was performed using a CMOS camera (30 fps/s, Basler, Ahrensburg, Germany), and the light source was controlled by an LED cold light source (6000–6500 K) under room temperature. Distilled water (1 μL) was dropped onto the surface of the films, which was measured in five different areas of each surface, and the average values were taken. Lastly, images of the droplets were taken by a camera.

### 2.9. Opacity (OP)

The transmittance of the composite films was measured using an ultraviolet spectrophotometer at 350–800 nm to determine the opacity of the composite films. Meanwhile, the opacity of the films was calculated based on the absorbance of the film samples at 600 nm. Each sample was measured three times, and the calculation formula was as follows:

(5) $$OP=\frac{A_{600}}{d}$$

where *OP* is the opacity, *A*_600_ is the absorbance at 600 nm, and *d* is the film thickness (mm). The higher the *OP* value, the higher the opacity and the lower the transparency.

### 2.10. FTIR

The dried film samples were cut into 30 × 30 mm and air dried by flushing with dry air to reduce the interference of H_2_O and CO_2_. After preparing KBr pellets at room temperature, the resolution of the FITR (1600, PerkinElmer Co., Waltham, MA, USA) was set to 2 cm^−1^ in the wavenumber range of 500–4000 cm^−1^. Infrared scanning was carried out 15 times to observe the chemical structure and group changes of the film samples and the infrared spectrograms of each sample were recorded.

### 2.11. XRD

The crystallinity of the composite films was tested using an X-ray diffractometer (D8-Advance, Bruker, NY, USA) according to a previously reported method with slight modifications [34]. All samples were measured using the X-ray diffractometer with a scanning angle of 5–80° (2θ), a voltage of 45 kV, and a current of 20 mA.

### 2.12. SEM

The dried films were cut into rectangles of 4 cm × 6 cm and dehydrated in liquid nitrogen, and gold was sprayed on the horizontal plane and cross-section of the films to be measured, with the samples being fixed on a table under a vacuum. The thickness was approximately 10 μm. The surface and cross-sectional images of the films were taken using a scanning electron microscope (Hitachi S4100, Tokyo, Japan), with voltages of 5.0 kV (surface) and 1.0 kV (cross-sectional) and magnifications of 10,000 × (surface) and 5000 × (cross-sectional) (Zeiss, Oberkohen, Germany).

### 2.13. Antibacterial Activity of CS, CS/LM, and CS/LM/SA

Each bacterial strain (*Escherichia coli*, *Shewanella putrefaciens*, *Staphylococcus aureus*, and *Salmonella typhimurium*) was coated onto 1.5% nutrient agar plates, and sterilized Oxford cups (6 mm) were placed on the agar [34]. We ensured that the bottom fit with the surface of the medium without gaps. Then, based on the results obtained (as described in Section 2.4), 50 μL of each solution (CS: 0.016 g/mL, CS/LM: 0.016/0.0015 g/mL, and CS/LM/SA: 0.016/0.0015/0.002 g/mL) was added into the Oxford cups. The plates were incubated overnight at 37 ± 2 °C for 36 h. The antibacterial activity was observed by measuring the diameter of the inhibition zone using a caliper. Each experiment was carried out in triplicate, and a 1% acetic acid solution was used as the control.

Meanwhile, the antibacterial activity of the films produced by CS, CS/LM, and CS/LM/SA was also detected; each film was cut into 6 mm diameters (according to Oxford cups), and the film was pressed by Oxford cups. Each experiment was carried out in triplicate, and a 1% acetic acid solution was used as the control.

### 2.14. Preservation Effect of Crayfish by Different Films

#### 2.14.1. Sample Preparation

After the crayfish arrived at the laboratory, crayfish of the same size were picked out and washed with clean water, and then the heads and crayfish lines were removed. Afterward, based on the results of Section 2.4, the crayfish were wrapped with different films by hand with sterile gloves in a sterile room; polyethylene (PE, 0.025 mm, 0.8 g·mm/m^2^·h·Pa, from Wushang Supermarket) was used as the control group, and the CS film, CS/LM film, and CS/LM/CA film were used as the experiment group. The wrapped crayfish can be seen in Figure S1, and all samples were put onto a plate and stored at 4 °C.

#### 2.14.2. Determination of Total Volatile Basic Nitrogen (TVB-N) and Total Aerobic Micro-organisms

The content of TVB-N in the crayfish was evaluated based on the national standard (GB5009.228–2016) of the People’s Republic of China and was determined using the automatic Kjeldahl nitrogen (Kjeltec-8400, Foss, San Francisco, USA) determination method. The crayfish were stripped from the films and then cleaned and drained in a sterile environment. A total of 5.0 g of crayfish meat was homogenized and extracted with 50 mL of water for 30 min and then centrifuged to remove the supernatant. MgO (1 g) was added, and the sample was transferred into a 750 mL digestive tube. A boric acid aqueous solution (20 g/L) was added to the distillate, and a mixture of methyl red and bromocresol green was used as an indicator for titration with 0.1 mol/L of hydrochloric acid. The grouped samples were tested three times in parallel.

The value of total aerobic micro-organisms was analyzed as follows: the preparation of the samples was the same as for TVB-N (mentioned above), but all processes were finished under sterile conditions, and the experimental materials were sterilized under a high temperature. The bacterial suspension was diluted using the 10× dilution method (PBS buffer at pH 7.0). The appropriate bacterial solution (0.1 mL) with three gradients was coated on the surface of the plate. The content of the total aerobic micro-organisms of the dilution was measured using the standard plate count method in nutrient agar after being incubated at 30 °C for 48 h, and the value of the total aerobic micro-organisms was expressed as log CFU/g for the crayfish [35].

#### 2.14.3. pH Analysis

For the determination of pH, 2.0 g of the sample was homogenized in 18.0 mL of distilled water for approximately 1 min, stood for 10 min, and centrifuged for 10 min at 6000× *g*. The pH of the sample supernatant was evaluated using a pH electrode (SIN-DC2000, Zurich, Switzerland).

#### 2.14.4. Thiobarbituric Acid Reactive Substance (TBARS) Assay

TBARS values were measured, as previously described [36]. A sample of 5.0 g of crayfish meat was homogenized with 25 mL of trichloroacetic acid (7.5% dint *w*/*w*). The homogenate was shaken at 50 °C on a constant temperature oscillator for 30 min and then filtered. Then, 5 mL of thiobarbituric acid reagent (1:1, *v*/*w*) was added into the filtrate and incubated at 90 °C for 40 min. Using a blank as the reference, the content of malondialdehyde (MDA) was determined at 532 nm using a spectrophotometer (U-2800, Thermo scientific, Boston, MA, USA). The TBARS values are expressed as mg MDA/kg.

#### 2.14.5. Texture Profile Analysis (TPA)

According to Pissia et al. [37], the TPA was determined using a Lab Pro texturometer (Food Technology Corp, Sterling, VA, USA) to analyze hardness, springiness, chewiness, and cohesiveness. Before the analyses, the samples were placed at room temperature for 1 h to reduce the effect of low temperature. The experimental conditions were as follows: P/5 plane cylindrical samples with a text speed of 1 mm/s, sample compression rate of 50%, and initial strength of 0.8 N. Each experiment was triple-replicated.

### 2.15. Statistical Analysis

All data were collated in Excel 2013, and the IBM-SPSS Statistics (20, IBM, Armonk, NY, USA) software was used for data variance and significant difference analysis. Design–Expert (7.16, State-East, San Diego, CA, USA) was used for the response surface methodology, and SPSS was used for regression analyses. One-way ANOVA was used for the analysis of variance, and the least significant difference method was used for the multiple comparison analysis (SPSS, IBM, Armonk, NY, USA) of the indicators with significant differences (*p* ≤ 0.05). The data are expressed as mean ± SD.

## 3. Results and Discussion

### 3.1. Irradiation of Chitosan

**Figure 1:**
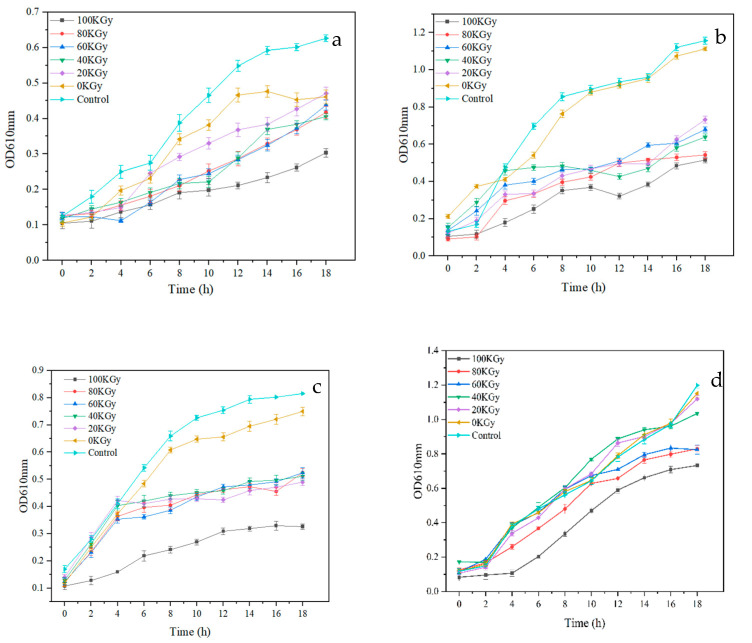
Irradiation under different doses of chitosan inhibition on *Escherichia coli* (**a**), *Shwanella putrefaciens* (**b**), *Staphylococcus aureus* (**c**) and *Salmonella typhimurium* (**d**).

Table 2 shows the molecular weight of the degraded chitosan after irradiation. The molecular weight of the nondegraded chitosan was very large (591,862 Da). After the γ-ray treatment, the relative viscosity of chitosan decreased significantly, and the molecular weight decreased at the same time. With increased radiation dose, the molecular weight of the chitosan decreased and remained basically unchanged after 100 KGy. This might be because the energy at this dosage could not break the chain of chitosan anymore [38]. Figure 1 shows the inhibitory effects of the irradiated chitosan at different doses on *Escherichia coli* (Figure 1a), *Shewanella putrefaciens* (Figure 1b), *Staphylococcus aureus* (Figure 1c), and *Salmonella typhimurium* (Figure 1d). Chitosan had the best antibacterial effect under a 100 KGy dose since it had the lowest molecular weight. The inhibitory effect on the four bacteria was as follows: *Staphylococcus aureus* > *Escherichia coli* > *Salmonella typhimurium* > *Shewanella putrefaciens*. This is also consistent with the previous report by Matsuhashi [39] in that 100 KGy-irradiated chitosan could inhibit the growth of *Escherichia coli*. After irradiation, irradiated chitosan exposes more active groups, such as amino acids and hydrogen. These groups more easily combine with negatively charged cell walls and make bacteria collapse [40]. Moreover, some studies proved that chitosan with a low molecular weight was more likely to enter the interior of bacteria and combine with DNA to disrupt cell proliferation [41]. This may be because chitosan can generate new active sites after irradiation, which subsequently scavenge free radicals. Therefore, we used 100 KGy-irradiated chitosan as the base research material.

### 3.2. Response Surface Experiment

From the results of the single-factor experiments (Figure S2), the TVB-N value of the crayfish decreased with an increase in chitosan concentration; this might be because the levels of antibacterial components and the density of the composite film increased with increased chitosan concentration. When the concentration of chitosan was higher than 0.016 g/mL, the preservation effect did not increase further since the thickness of the composite film and its moisture permeability coefficient increased with higher concentrations of chitosan [42]. For lysozyme, the composite film showed the best preservation performance at 0.0015 g/mL; this might be because a higher concentration of lysozyme reduces the light transmittance of the film, resulting in worsened preservation. Additionally, the best concentration of carrageenan was 0.002 g/mL.

The response surface method (RSM) results are shown in Table S1; the quadratic multiple regression equation is as follows: TVB-N = 23.73 − 1.64A + 0.015B − 0.27C − 0.98AC + 0.78BC + 1.93A^2^ + 3.98B^2^ + 2.17C^2^ (A:CS, B:LM, and C:CA). The *p*-value of the regression model is <0.01, indicating statistical significance. Moreover, the *p*-value for lack of fit is >0.05, which supports the statistical significance. The correlation coefficient R^2^ is 0.9844, and the R^2^ Adj is 0.9644, indicating that this model could explain 96.44% of the response value changes and that the equation fits well. The order of influence of the selected factors on TVB-N is as follows: CS > LM > CA; furthermore, CS has a significant effect on TVB-N. The *p*-values for A^2^, B^2^, and C^2^ suggest that the relationship between the factors and TVB-N is not simply linear and that the quadratic term also has a great influence on TVB-N. Lastly, the RSM results reveal that the optimum conditions are CS at 0.016 g/mL, CA at 0.0019 g/mL, and LM at 0.0025 g/mL. The calculated results are almost equal to those of the single-factor experiments, and after considering the operability, the final components were set as follows: CS: 0.016 g/mL; CA: 0. 0.0015 g/mL; and LM: 0.002 g/mL.

### 3.3. Tensile Strength and Elongation Break of Different Films

The mechanical fracture resistance and tensile degree of the composite films were evaluated based on TS and EB [43]. Generally speaking, the mechanical properties of the composite films changed with changes in the corresponding composition. Consistent with the results of El-Fawal [44], a significant increase in TS was observed after the incorporation of CA into the composite films, and this result was mainly due to the fact that CS could combine with CA through electrostatic attraction (hydrogen and hydroxyl) and then increase the TS of the composite films [45] (Table 3). At the same time, the elongation at the break of the composite films increased, and the films became more elastic.

### 3.4. Physical Characteristics of Different Composite Films

From our results, the thickness of the films increased with the addition of carrageenan, which might be due to an increase in the content of film-forming materials in the composite films. For food preservation, water vapor permeability is very important since the lower the water vapor transmission rate is, the better the barrier performance [46]. As shown in Table 3, chitosan shows low water barrier performance because of its high hydrophilicity, and the addition of lysozyme has no significant effect on the water vapor barrier performance of the films; these results are consistent with the results of Park [47]. With the addition of carrageenan, the water vapor permeability of the CS/LM/CA film decreased, which might be because, with the addition of CA, crystallinity increased or because free hydrophilic groups (OH and NH) reduced when water vapor flowed through the film [48]. Moreover, the film properties, such as WVTR and TS, can be affected by film thickness based on Equation (4) and the equation for TS from other studies [49]. In our results, when comparing the thickness of the films and the WVTR and TS values, it could be observed that these values are affected by the properties of the additives more than film thickness.

The results of the oxygen permeability of the films are shown in Table 3; the oxygen permeability of the CS film was small (31.66 cm^3^·µm/m^2^·day·kPa), which means it has a good ability to stop the transmission of oxygen. Meanwhile, the CS/LM composite film was almost the same as the CS film in terms of the opacity results. With the addition of CA, the oxygen permeability of the composite film (CS/LM/CA) increased (24.31 cm^3^·µm/m^2^·day·kPa), and this might be because the cross-linked structure formed by CS and CA increased the density (based on our SEM studies).

### 3.5. Color Analysis

The color of the composite films is very important since it can affect the appearance of food. The color difference was determined using three parameters: L, a, and b. After adding lysozyme, the color parameters of the films were different (Table 4). The ΔE value only slightly increased by 0.5 with the addition of lysozyme, which indicated that the addition of lysozyme did not affect changes in film color. When compared to the CS/LM/CA film, the L value obviously decreased, which indicated that the film darkened, while increased a and b values indicated that the film had turned red and yellow. The above results showed that with increased carrageenan content, the film color became darker and more yellow and reddish (Figure S3). The reason is that, in our research, the solution of LM is clear and cannot affect the color of a film, while CA has a yellowish tint, which could contribute to color changes [50].

### 3.6. Opacity

The opacity of composite films is an issue of great concern in the development of food packaging materials. Ultraviolet and visible light irradiation accelerates fat deterioration, leading to the discoloration and deterioration of products. Previous studies have shown that increasing the opacity of food packaging films can reduce and prevent food exposure to the sun, thus decreasing fat deterioration. As shown in Table 4 and Figure S3, the opacity of the chitosan–lysozyme composite film is similar to the chitosan film, which might be because the size of lysozyme nanoparticles is smaller than the visible wavelength. At the same time, the opacity of the CS/LM/CA composite film increased, indicating that the barrier performance of the composite film to visible light has improved [51].

### 3.7. Water Contact Angle

**Figure 2:**
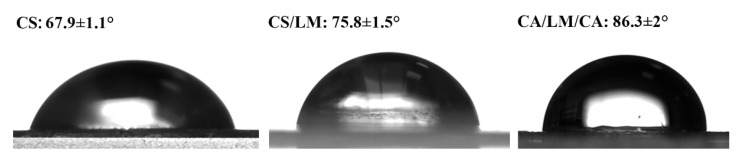
Water contact angle of the different composite films.

Water contact angle is an important index to evaluate the hydrophilic/hydrophobic properties of films [51]. Usually, a low contact angle (<90°) indicates hydrophilicity, and a high contact angle (>90°) indicates hydrophobicity. The contact angle of the composite films is shown in Figure 2; the water contact angle of all groups was less than 90°, indicating that all the composite films were hydrophilic. The contact angle of the irradiated chitosan film was 68°, and the droplets were completely absorbed into the chitosan composite film after 1 min. This is due to the high hydrophilicity and wettability of the surface of the chitosan chain, arising from its rough surface and hydrophilic skeleton [43]. However, the contact angle of the CS/LM composite film was 75°; this might be due to the existence of hydrophobic amino groups with a special structure in lysozyme, which increased the contact angle [48]. When compared with the pure chitosan film, the contact angle of the CS/LM/CA composite film increased by 19°; this might be because carrageenan increased the surface roughness of the composite film (as can be seen in the SEM image) [51]. According to other studies, the water contact angle is positively correlated with material roughness, as reported in the study by Shahbazi [52].

### 3.8. FTIR Analysis

**Figure 3:**
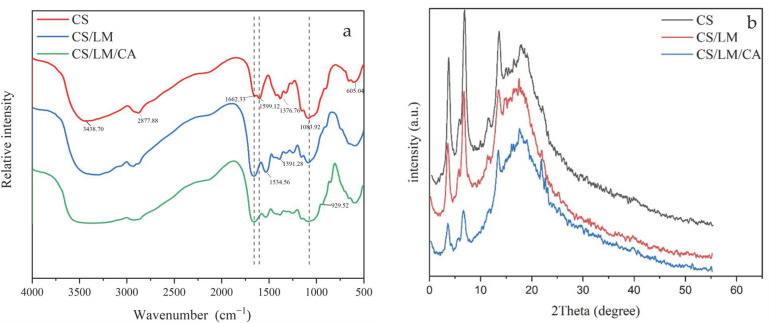
FT-IR (**a**) and XRD (**b**) of different composite films.

FTIR is an important tool that is used to identify affected functional groups in macromolecules. We performed FTIR analysis to examine the effects of lysozyme and carrageenan on the functional groups in our composite films. As shown in Figure 3a, the wide peak at 3438.70 cm^−1^ of the simple chitosan film is mainly composed of multiple absorption peaks formed by the overlap of -NH and -OH, which indicates that these amino and hydroxyl groups have different intramolecular and intermolecular hydrogen bonds. The peak at 2877.88 cm^−1^ represents the anti-symmetric stretching vibration absorption peak of -CH_3_, and the C=O stretching (amide Ι band) and N-H stretching (amide II band) bending vibration peaks appear at 1662.33 cm^−1^ and 1599.12 cm^−1^, respectively. The peaks at 1376.76 cm^−1^ and 1089.92 cm^−1^ are associated with C-N stretching vibration generation (amide III band) and the meso-hydroxy (C_3_-OH) C-O stretching vibration absorption peak, respectively. These typical infrared characteristic peaks of chitosan films observed in the composite film are consistent with previous reports [52,53]. When compared to the spectrum of the composite film with lysozyme, the infrared spectrum curve of the composite film with lysozyme and carrageenan does not change greatly, which might be related to the amount of lysozyme added. The absorption peak of the carrageenan composite film near 929 cm^−1^ indicates that carrageenan contains a sulfuric acid group. Overall, the results of the FTIR analysis suggest that lysozyme and carrageenan have been successfully mixed into the composite film.

### 3.9. XRD Analysis

The X-ray diffraction pattern of the chitosan composite film is shown in Figure 3b, which reveals the crystallinity of the polymer. Chitosan is a partially crystalline polysaccharide with four polymorphisms: “tendon,” “form II,” “annealed,” and “1–2.” [53,54]. The chitosan film has a weak peak at approximately 8° and a sharp peak at 12°, which correspond to the “type II” polymorphism of chitosan acetate and the “annealed” crystal of chitosan, respectively; these results are consistent with a previous report by Anglin et al. [55]. The peak at 18° may also be caused by the presence of chitosan acetate. There is no change in the XRD pattern of the composite film after adding lysozyme, which indicates that the lysozyme and chitosan were well fused or that their crystallinity was poor. However, the addition of carrageenan led to a decrease in the peak width and peak area, indicating that the addition of carrageenan affects the crystallinity of chitosan.

### 3.10. SEM Analysis

**Figure 4:**
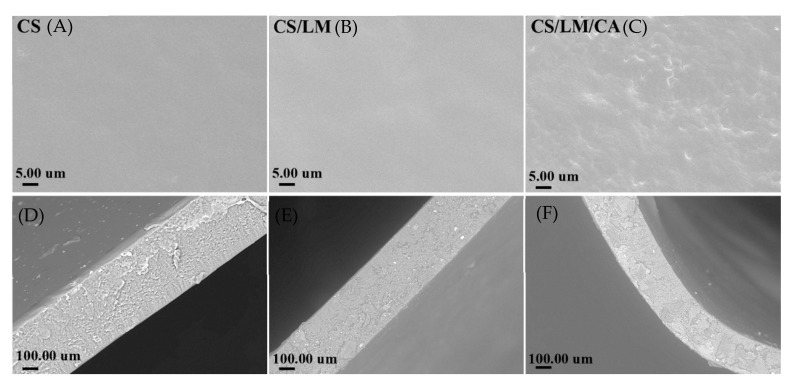
Scanning electron microscopy (SEM) images of the surface and cross-section of composite film. Surface: (**A**) CS; (**B**) CS/LM; (**C**) CS/LM/CA (5000× magnification); cross-section: (**D**) CS; (**E**) CS/LM; (**F**) CS/LM/CA (1000× magnification).

The microstructure of the composite films is shown in Figure 4. The surface of the pure chitosan film was smooth and flat without holes and cracks, and its cross-sections also showed a fine and uniform texture. When lysozyme was added, it was easily soluble in water and could be uniformly dispersed in the chitosan solution; thus, the surface of the chitosan–lysozyme composite film was smooth and flat. With the addition of carrageenan, the surface of the film became rougher, but there were no cracks and pores. Moreover, from the results of the transverse sections, there were several hollowed structures in the chitosan film, indicating that its mechanical performance was poor, which is consistent with the TS and EB results. With the addition of carrageenan, the composite film became tighter, and the hollowed structure was reduced. Thus, the CS/LM/CA composite film had good mechanical properties.

### 3.11. Antibacterial Activity

**Figure 5:**
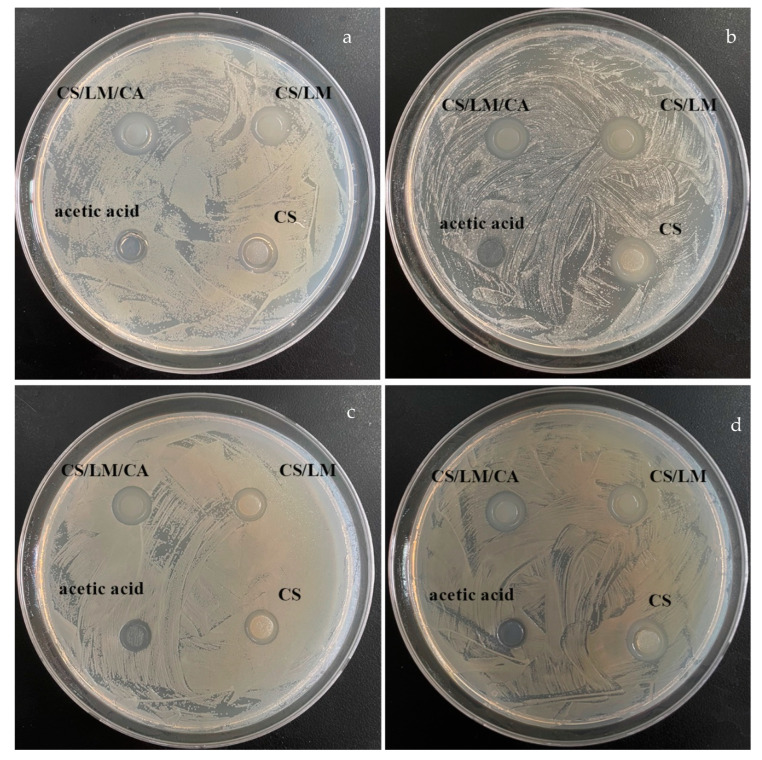
Oxford cup diffusion antibacterial test for CS, CS/LM, CS/LM/CA, and 1% acetic acid solution against bacterial strains: (**a**) *Salmonella typhimurium*, (**b**) *Staphylococcus aureus*, (**c**) *Shewanella putrefaciens*, and (**d**) *Escherichia coli*.

The antibacterial activity of the CS, CS/LM, and CS/LM/CA solutions was carried out against *Escherichia coli*, *Shewanella putrefaciens*, *Staphylococcus aureus*, and *Salmonella typhimurium* using the Oxford cup assay. The zones of inhibition produced by the CS, CS/LM, CS/LM/CA, and 1% acetic acid solutions are presented in Figure 5, and the comparative values are shown in Table 5. The magnitude of antibacterial ability of the solutions is as follows: 1% acetic acid < CS < CS/LM < CS/LM/CA, and the inhibition zones are 7–8, 9–11, 13–14, and 14–16 mm, respectively. Moreover, the results of the zone of inhibitions suggest that both LM and CA can increase the antibacterial activity of the films, but the effect of LM is more obvious due to the fact that lysozyme is a muramidase and thus, it can hydrolyze cell walls and make bacteria autolyze [55]. The results of the antibacterial activity of the films produced by CS, CS/LM, and CS/LM/CA are presented in Figure S4; the zones of inhibition of the films are almost similar to those of the solutions, which suggests that drying would not decrease the antibacterial activity of CS, CS/LM, and CS/LM/CA, and the CS/LM/CA composite film still has the highest antibacterial activity.

### 3.12. Quality Changes in Crayfish Meat during Storage

#### 3.12.1. TVB-N and Total Aerobic Micro-Organisms

**Figure 6:**
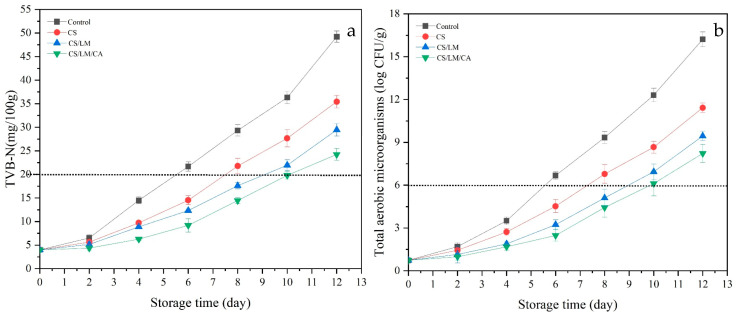
Changes in TVB-N (**a**) and total aerobic micro-organism (**b**) values of crayfish packaged with different films under 4 °C.

The accumulation of TVB-N may be due to the decomposition of nitrogen-containing substances, such as crayfish meat protein, under the action of endogenous enzymes and micro-organisms to produce ammonia, amine, and other alkaline substances [56]. When the TVB-N value is ≥20 mg/100 g, the products can be considered to have been corrupted [53]. The TVB-N of the control group was 3.98 ± 0.14 mg/100 g on day 0, and the TVB-N value reached 21.25 ± 1.25 mg/100 g on day 6 (Figure 6a), which was beyond the acceptable range. The rising trend in TVB-N of the control group was significantly higher than that of the treatment group. For crayfish packaged with the composite fresh-keeping film, during the whole storage period, the TVB-N of the treatment group increased slowly (Figure 6a), and the TVB-N of the CS/LM/CA film exceeded the acceptable range on the 10th day (20.81 ± 0.87 mg/100 g). Meanwhile, the shelf life of the CS and CS/LM films was around 7 and 9 days, respectively. This might be because the PE film had poor antibacterial properties, and the hydrophobic properties of the CS and CS/LM films were lower than the CS/LM/CA film. The total aerobic micro-organisms reflected the same results. When the value of the total aerobic micro-organisms is higher than 6 log CFU/g, this means that the packaged crayfish are incredibly spoiled [57]. On the 6th day (Figure 6b), the total aerobic micro-organism value of the control group was 6.51 ± 0.23 log CFU/g, and the LM/CA group exceeded 6 log CFU/g on the 10th day (6.11 ± 0.35 log CFU/g). Thus, the CS/LM/CA group had the best fresh-keeping effect and could extend the shelf life of crayfish up to 10 days at 4 °C.

#### 3.12.2. pH

**Figure 7:**
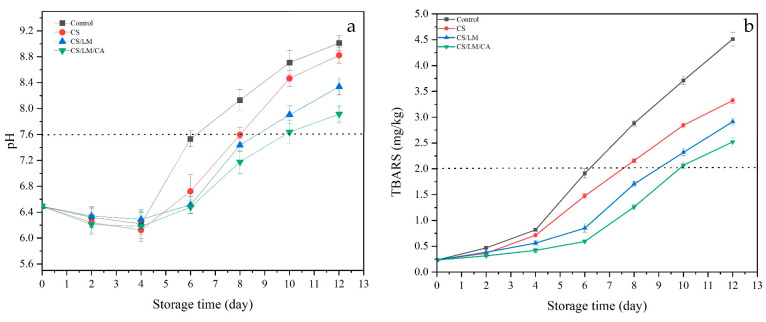
Changes in pH (**a**) and TABRS (**b**) values of crayfish packaged with different films under 4 °C.

The pH of all groups decreased at first and then increased (Figure 7a). During the initial period, the pH value decreased, which might be due to the accumulation of lactic acid and the degradation of adenosine triphosphate to release inorganic phosphate as a result of the glycolysis of crayfish meat [58]. With more storage time, the pH gradually increased, mainly due to the decomposition of proteins into nitrogen-containing basic compounds, such as amino acids, trimethylamine, and indole, by the micro-organisms and endogenous enzymes in crayfish [59]. Normally, when the pH is higher than 7.6, the packaged crayfish can be considered unacceptable. During storage, when compared to the control group, the pH of the composite films increased slowly, and the pH of the CS/LM/CA film exceeded 7.6 on day 10; this might be because the composite film effectively inhibited the autolysis process catalyzed by endogenous enzymes, thus slowing down the production of basic compounds [58].

#### 3.12.3. TBARS Assay

Fat oxidation can be caused by enzymatic or non-enzymatic reactions, which can lead to the spoilage of crayfish meat during storage. The degree of fat degradation in meat products can be expressed using the TBARS value, which reflects the quality of meat during storage, and the value of TBARS cannot exceed 2 [59]. The TBARS value of the crayfish meat in the control group and other groups presented an upward trend with extended storage time (Figure 7b). The initial value of each sample was 0.27 ± 0.04 mg/kg, and the control group increased significantly with storage time, reaching 1.95 ± 0.05 mg/kg on day 6. However, the TBARS value of the CS/LM/CA group increased from 0.27 ± 0.04 mg/kg to 2.04 ± 0.08 mg/kg on day 10. This is mainly due to the inhibition of microbial growth by chitosan and lysozyme, which reduces the content of the lipase produced by microbial metabolism and prevents the decomposition of fat [60].

#### 3.12.4. Texture Profile Analysis

**Figure 8:**
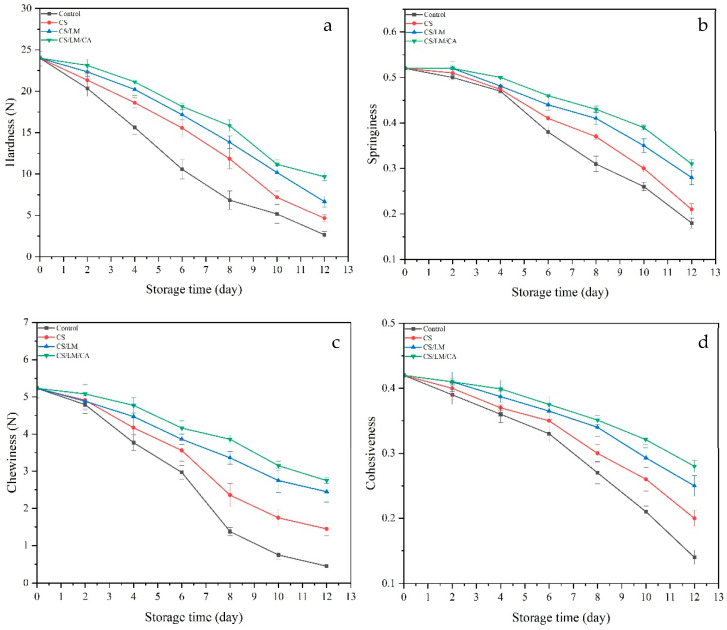
Changes in TPA of crayfish packaged with different films under 4 °C: (**a**) Hardness; (**b**) Springiness; (**c**) Chewiness; (**d**) Cohesiveness.

The TPA results are shown in Figure 8, including hardness (a), springiness (b), chewiness (c), and cohesiveness (d). During storage, the values of the TPA results decreased. This might be because the TPA could be related to the diameter of muscle fibers and the contents of free water and myofibril attachment [61]. From the results, we could observe that during the early stage, the declining rate of all groups was slow, especially for hardness, and the rate decreased rapidly with a longer time. This might be because low temperatures could control water vapor spread from the crayfish muscle, and these films had the ability to prevent water vapor transmission [62]. With longer storage time, spoilage bacteria increased, especially for the PE film. This may be because the myofibrillar protein was degraded, muscle fiber bundles became damaged, and the muscle fibers were broken and loose, resulting in a decreasing TPA [63].

## 4. Conclusions

In this study, we successfully fabricated a fresh-keeping composite film incorporating modified CS, CA, and LM. The modified CS was degraded by gamma-ray irradiation and had the best antibacterial ability under the 100 KGy dose; this might be because chitosan can generate new active sites after irradiation, which subsequently scavenge free radicals. With the addition of CA and LM, the mixture could form hydrogen bonds and other groups [64], which improved the physical and mechanical properties of the composite film; the tensile strength increased by 20.2%, the elongation at break increased by 56.6%, and hydrophobicity increased by 19°. Furthermore, the CS/LM/CA composite film demonstrated a good protection effect for crayfish, maintaining lower pH, TBRAS, and TPA values due to its good antibacterial activity and WVTR. The changes in TVB-N and total aerobic micro-organism values indicated that the shelf life of crayfish meat could be extended up to the 10th day of storage at 4 °C by the CS/LM/CA film. Therefore, the fabricated CS/LM/CA composite film has the potential to improve the meat quality and shelf life of crayfish during storage.

## Figures and Tables

**Table 1:** The concentration of materials in the films.

Material	Concentration (g/mL)				
CS	0.001	0.015	0.02	0.025	0.03
LM	0.001	0.0015	0.002	0.0025	0.003
CA	0.0005	0.001	0.0015	0.002	0.0025

**Table 2:** Molecular weight at various irradiation doses.

Irradiation Dose	0 KGy	20 KGy	40 KGy	60 KGy	80 KGy	100 KGy
Intrinsic viscosity	380	237	127	115	111	110
molecular weight	591,862	329,097	151,527	133,937	128,170	126,736

**Table 3:** Thickness, tensile strength (TS), elongation break (EB), and water vapor permeability of different composite films.

Content	Thickness (mm)	TS (N)	EB (%)	WVTR (g·mm/m^2^·h·Pa)	Oxygen Permeability (m^3^·µm/m^2^·day·kPa)
CS	0.077 ± 0.004 ^b^	16.87 ± 0.78 ^b^	3.83 ± 0.01 ^b^	5.83 ± 0.39 ^a^	31.66 ± 0.94 ^b^
CS/LM	0.078 ± 0.001 ^b^	17.46 ± 0.64 ^b^	4.35 ± 0.08 ^b^	5.74 ± 0.14 ^a^	30.14 ± 0.98 ^b^
CS/LM/CA	0.112 ± 0.002 ^a^	20.28 ± 0.18 ^a^	6 ± 0.32 ^a^	4.75 ± 0.11 ^b^	24.31 ± 0.81 ^a^

Note: different letters in each column indicate significant differences between groups via the LSD test.

**Table 4:** Color and opacity parameters of different composite films.

Films	ΔE	*L*	a	b	W	*OP* (mm^−1^)
CS	8.73 ± 0.49 ^b^	−2.67 ± 0.38 ^a^	−0.37 ± 0.06 ^a^	8.30 ± 0.44 ^b^	−3.01 ± 0.41 ^a^	4.85 ± 0.29 ^b^
CS/LM	9.23 ± 0.41 ^b^	−2.83 ± 0.32 ^b^	−0.43 ± 0.05 ^a^	8.80 ± 0.36 ^b^	−3.21 ± 0.35 ^b^	4.7 ± 0.19 ^b^
CS/LM/CA	18.5 ± 0.52 ^a^	−6.53 ± 0.25 ^a^	0.60 ± 0.26 ^b^	17.27 ± 0.49 ^a^	−7.93 ± 0.31 ^b^	8.59 ± 0.25 ^a^

Note: data are expressed as mean ± SD. Values in each column with different lowercase letters indicate a significant difference between groups via the LSD test.

**Table 5:** Zone of inhibition values for CS, CS/LM, CS/LM/CA, and 1% acetic acid solution against bacterial strains.

Bacterial Strains	Zone of Inhibition (mm)			
	1% Acetic	CS	CS/LM	CS/LM/CA
*Salmonella* *typhimurium*	8.37 ± 0.24 ^a^	10.04 ± 0.13 ^b^	13.74 ± 0.12 ^ab^	14.25 ± 0.66 ^a^
*Shewanella* *putrefaciens*	7.59 ± 0.30 ^b^	9.97 ± 0.29 ^a^	14.32 ± 0.31 ^a^	14.78 ± 0.21 ^a^
*Staphylococcus* *aureus*	8.34 ± 0.15 ^a^	11.18 ± 0.76 ^b^	14.19 ± 0.85 ^b^	16.37 ± 0.12 ^a^
*Escherichia coli*	8.21 ± 0.12 ^a^	10.36 ± 0.12 ^ab^	12.01 ± 0.59 ^ab^	14.49 ± 0.11 ^a^

Data are expressed as mean ± SD. Values in each column with different lowercase letters indicate a significant difference between the groups via the LSD test.

## Data Availability

The data used to support the findings of this study are available from the corresponding author upon request.

## Supplementary Materials

The following supporting information can be downloaded at: https://www.mdpi.com/article/10.3390/foods12142642/s1. Figure S1. Crayfish wrapped with different films (PE,CS,CS/LM,C/LM/CA); Figure S2 The results of single factor experiment (a: CS; b: LM; c: CA); Table S1. Response Surface Regression Equation Analysis of Variance; Figure S3. Color of different composite films (a: CS film, b: CS/LM film; c: CS/LM/CA film; d: Transmissivity); Figure S4. Oxford cup diffusion antibacterial test of CS, CS/LM, CS/LM/CA films and 1% acetic acid solution against bacterial strains, (a) *Salmonella typhimurium* (b) *Staphylococcus aureus* (c) *Shewanella putrefaciens* (d) *Escherichia coli*.
