# Supplementary material for: Preparation and Characterization of a Biodegradable Film Using Irradiated Chitosan Incorporated with Lysozyme and Carrageenan and Its Application in Crayfish Preservation
Source: Foods. 2023 Jul 8;12(14):2642. doi: 10.3390/foods12142642 (PMC10378868; doi:10.3390/foods12142642)
Supplement: Supplementary file 1 [file foods-12-02642-s001.zip › foods-2368844-supplementary.pdf]

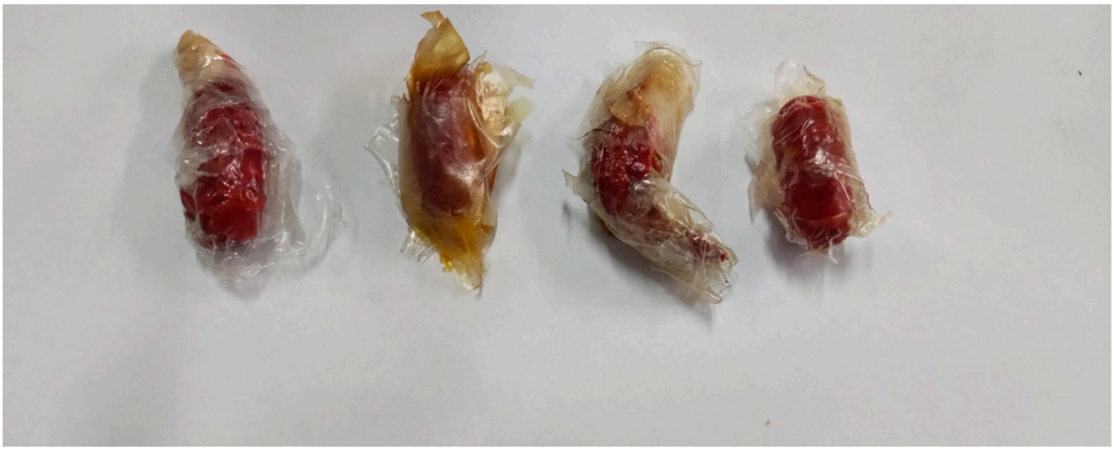

FigureS1. Crayfish wrapped with different films (PE,CS,CS/LM,C/LM/CA)

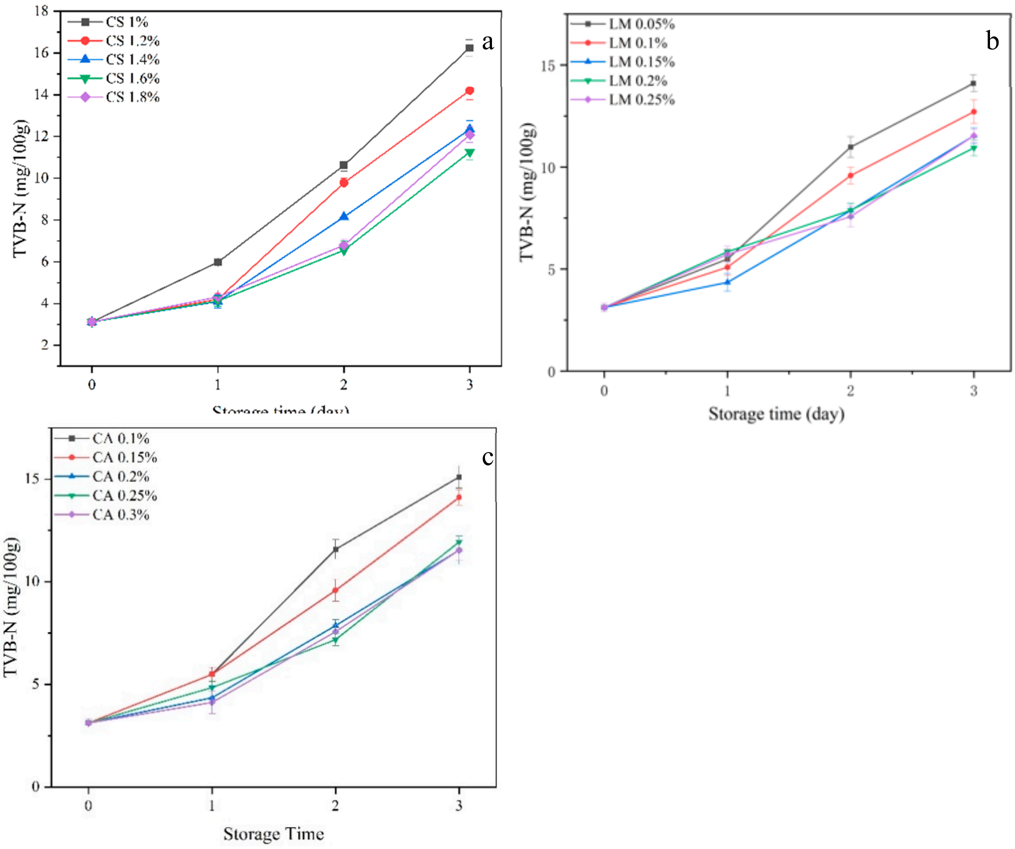

Figure S2. The results of single factor experiment (a: CS; b: LM; c: CA)

Table S1. Response Surface Regression Equation Analysis of Variance

Source	Sum of squares	df	Mean square	F value	P value
Model	141.00	8	17.62	18.97	<0.0001
A-CS	21.64	1	21.64	8.77	<0.0001
B-CA	4.61	1	4.61	3.34	0.6414

C-LM	0.59	1	1.26	1.91	0.2044
AC	3.88	1	3.86	12.53	0.0076
BC	2.45	1	64.00	7.92	0.0227
A^2	15.61	1	42.74	50.45	0.0001
B^2	66.79	1	84.45	215.83	<0.0001
C^2	19.78	1	3.63	63.93	<0.0001
Residual	2.48	8	1.38		
Lack of fit	1.04	4	0.9228	0.72	0.61902
Cor total	143.47	16			

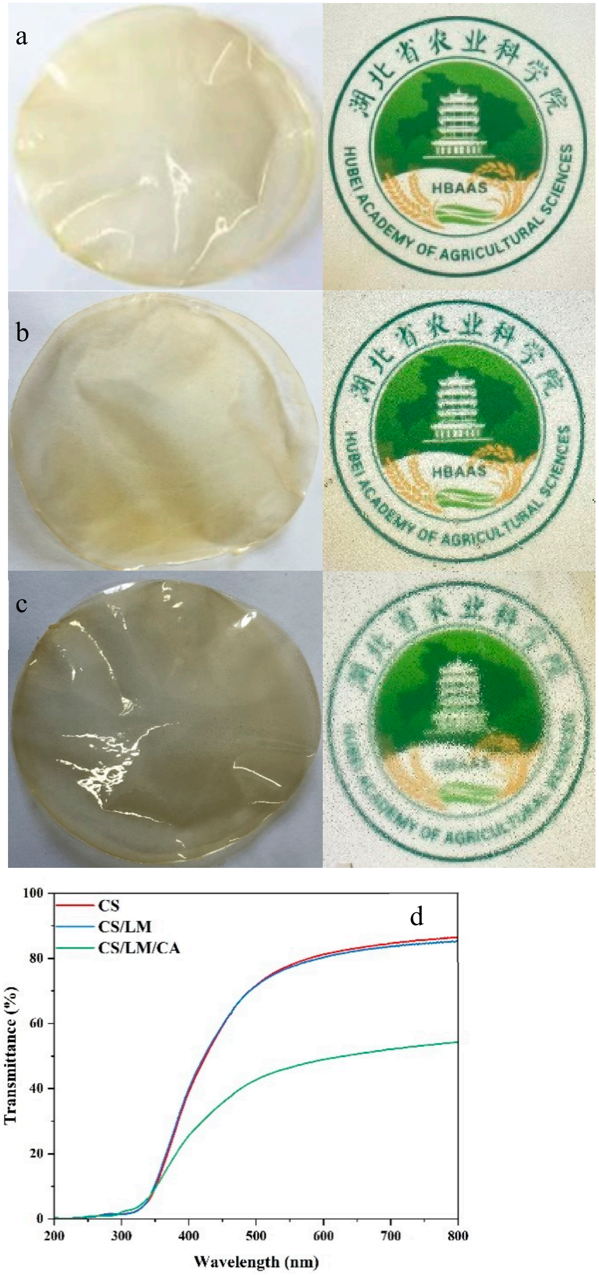

Figure S3. Color of different composite films (a: CS film, b: CS/LM film; c: CS/LM/CA film; d:

Transmissivity)

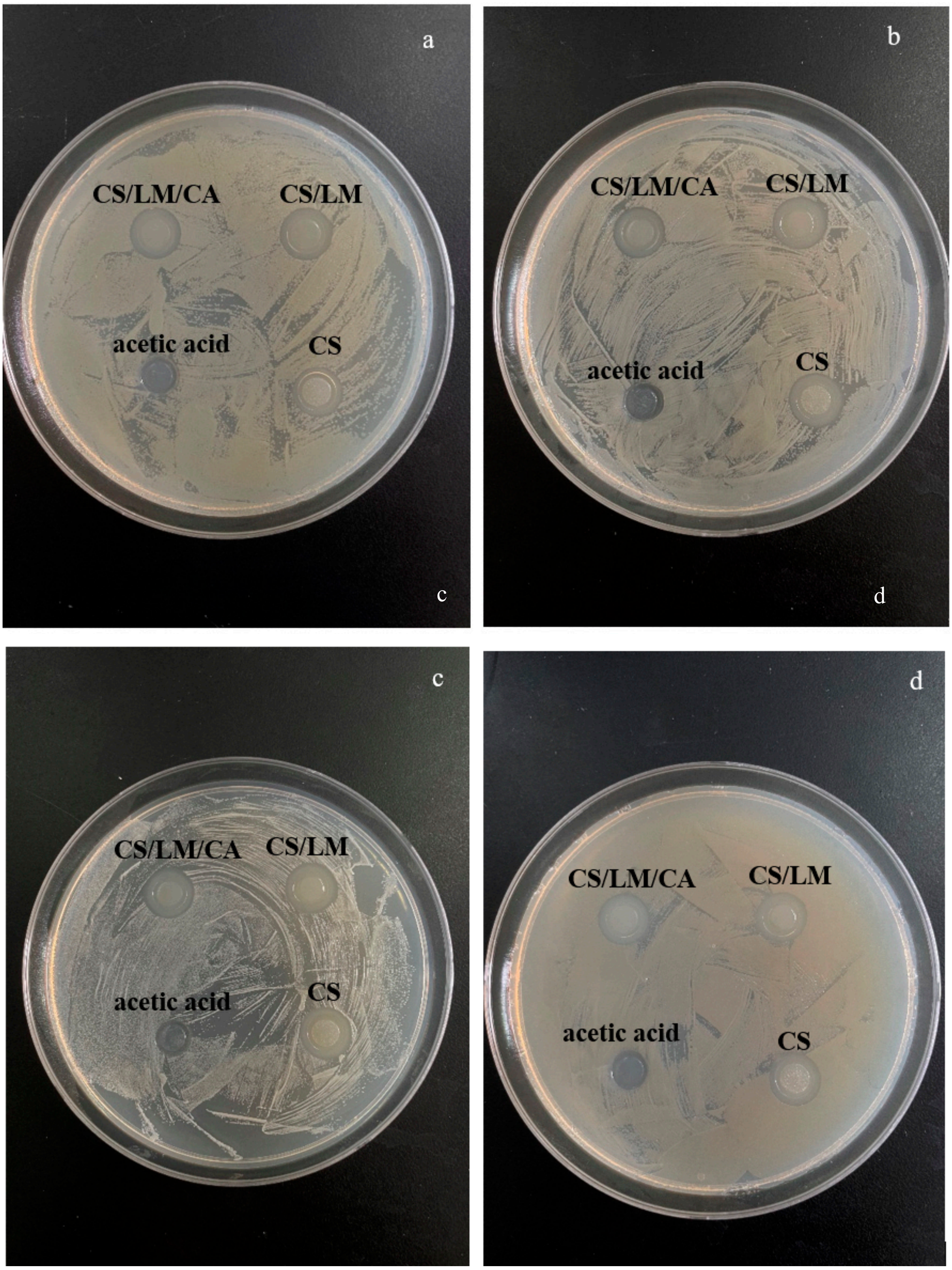

Figure S4. Oxford cup diffusion antibacterial test of CS, CS/LM, CS/LM/CA films and 1% acetic acid solution against bacterial strains, (a) Salmonella typhimurium (b) Staphylococcus aureus (c) Shewanella putrefaciens (d) Escherichia coli
